# Residual efficacy of indoor residual spraying using clothianidin (SumiShield 50WG) under experimental huts and field conditions in Senegal

**DOI:** 10.1186/s12936-025-05703-0

**Published:** 2026-01-16

**Authors:** Oumar Ciss, Abdoulaye Niang, Ousmane Sy, El Hadji Diouf, Moussa Diallo, Moussa Diop, Moussa Fall, Assane Ndiaye, Omar Thiaw, Babacar Ndiouck, Moussa Diagne, Malick Diouf, Ousmane Faye, Lassana Konate, El Hadji Amadou Niang

**Affiliations:** 1 Laboratory of Vector and Parasite Ecology of Cheikh, Anta Diop University, Dakar, Sénégal; 2 Medical Research Council Unit, The Gambia at the London School of Hygiene and Tropical Medicine Atlantic Boulevard, Fajara, Kanifing Municipality The Gambia; 3 Department of Animal Biology, Cheikh Anta Diop University of Dakar, Dakar, Senegal

**Keywords:** Residual efficacy, IRS, Clothianidin, (SumiShield 50WG), Experimental huts, Field conditions, Senegal

## Abstract

**Background:**

In Senegal, the main vector control strategies include indoor residual spraying (IRS) and the distribution of insecticide-treated nets (ITNs). However, drugs and insecticides resistance have become a major challenge in the fight against malaria transmission. Addressing the problem of escalating resistance is vital to maintaining progress towards malaria elimination, which has stalled in recent years. New formulations belonging to the neonicotinoid class, clothianidin, have been developed and is now being used for malaria vector control through IRS.

**Methods:**

The residual efficacy of clothianidin-treated walls was assessed monthly using WHO cone bioassays. Five houses in each of the two treated villages were evaluated, while one untreated house served as a control. In the experimental huts, a total of six huts, three in banco (mud) and three in cement, were evaluated on a monthly basis. Three cones were installed on three walls of each sprayed house at heights of 0.5 m, 1 m and 1.5 m above the ground, and three additional cones were placed in the control house. Ten female *Anopheles coluzzii* mosquitoes, aged between 3 and 5 days and derived from a laboratory-susceptible strain, were exposed in each cone for 30 min. After exposure, the mosquitoes were transferred to cups and mortality rates were recorded up to four days after exposure.

**Results:**

Results demonstrate high efficacy of SumiShield 50WG on mud and cement substrates, residual activity for up to twelve months in experimental huts and eight months under field conditions. In experimental huts, the 96 h mortality rate of the susceptible mosquito strain remained at 100% throughout the study, except in months ten and twelve for mud-walled huts, and months six and ten for cement-walled huts, where mortality rates were 98.33%, 99.16%, 95.68%, and 97.52%, respectively. In the field sites of Bandafassi and Tomboronkoto, the 72 h mortality rate of the susceptible strain remained consistently at 100% over the eight-month period.

**Conclusions:**

Clothianidin, a neonicotinoid insecticide, has not yet shown resistance in malaria vectors in Senegal. SumiShield 50WG is effective for resistance management through a rotation strategy using insecticides with different modes of action across spray cycles.

## Background

Malaria remains a significant global public health issue. Paradoxically, this preventable and treatable disease remains a leading cause of death and a major contributor to poverty, particularly in Afrotropical countries. In 2024, nearly half of the world’s population was at risk of malaria, with the majority of cases and deaths occurring in the sub-Saharan Africa World Health Organization (WHO) region [1]. The 2024 World Malaria Report estimated that there were 246 million cases of malaria, resulting in 569,000 deaths. The WHO African region continues to bear a disproportionate burden, accounting for 94% of malaria cases and 95% of deaths [1].

Over the past two decades, the large-scale implementation of long-lasting insecticidal nets (LLINs), and indoor residual spraying (IRS) has significantly reduced malaria related morbidity and mortality across sub-Saharan Africa. However, the elimination of malaria requires a comprehensive strategy that integrates vector control measures with rapid diagnosis and treatment [2]. To improve vector control impact and preserve pyrethroids for insecticide-treated nets (ITNs), African IRS programmes have partially suspended the use of pyrethroids and organochlorines in favour of carbamate and organophosphates [3, 4]. Although these insecticides have proven highly toxic to malaria vectors, the short residual efficacy of their original formulations for indoor residual spraying was insufficient, necessitating the development of long-lasting alternatives. Among the most promising long-lasting formulation candidates, the microencapsulated formulation of pirimiphos-methyl which was developed (Actellic 300CS), demonstrated prolonged activity against pyrethroid-resistant malaria vector mosquitoes for up to 9 months [5, 6]. More recently, the use of molecules with a different mode of action, such as clothianidin that is particularly effective against many species of mosquitoes that have already developed resistance to one or even all major classes of insecticides currently available for IRS. This would allow substantial control of mosquito vectors and malaria across different eco-epidemiological settings. The neonicotinoid (clothianidin) has interesting potential for malaria vector control through IRS. Successful laboratory and field trials have led to the development of SumiShield 50WG [7].

In Senegal, malaria is endemic with the entire population at risk of the disease. The number of suspected and confirmed cases in 2020 was 452,984 [4]. Malaria transmission is mainly closely related to the rain regimen and season which often drives the vector population density trend. The southern part of Senegal, representing 11.3% of the country’s total population and consisting of the regions of Kedougou, Tambacounda and Kolda, is the red zone of high malaria transmission. In 2020, this zone recorded 83.3% of the malaria cases causing 43.6% of deaths of all ages and 78.8% of deaths of children under 5 years of age [8]. In order to reduce these alarming indicators, the National Malaria Control Programme (NMCP) decided to implement, in addition to long-lasting insecticide-treated mosquito nets (LLINs), targeted indoor residual spraying (IRS) in specific health districts in the centre and south-eastern parts of the country, in particular the district of Kedougou, which is one of the most malaria endemic areas in Senegal. The use of IRS as a large-scale vector control strategy began in Senegal in 2007 with the use of pyrethroids in the health districts of Richard-Toll, Nioro and Vélingara. Following the appearance of resistance to these molecules, a carbamate, the bendiocarb (Ficam^®^ WP 10) was introduced between 2011 and 2015. Then an encapsulated formulation of pirimiphos-methyl (organophosphate), namely the Actellic 300 CS, was first used in a pilot study in the Bambey, Mbour and Fatick districts in 2013 [9] and then as a replacement for bendiocarb in other parts of the country between 2014 and 2017. After being suspended in 2018, IRS activities restarted in 2019 and 2020 in the north and the south-east of the country. In 2020, two new Clothianidin-based formulations (SumiShield 50WG and Fludora Fusion) were introduced in vector control interventions. Before its use in the field, SumiShield 50WG was studied under semi-field conditions at the Ndioukhane station.

In this context of the use of this new insecticide formulation in national vector control strategies, the aim of the study is to evaluate and compare the residual efficacy of Clothianidin (SumiShield 50WG) both under the experimental huts condition in Ndioukhane field station then in operational IRS conditions in Kedougou.

## Methods

### Study areas

**Fig. 1:**
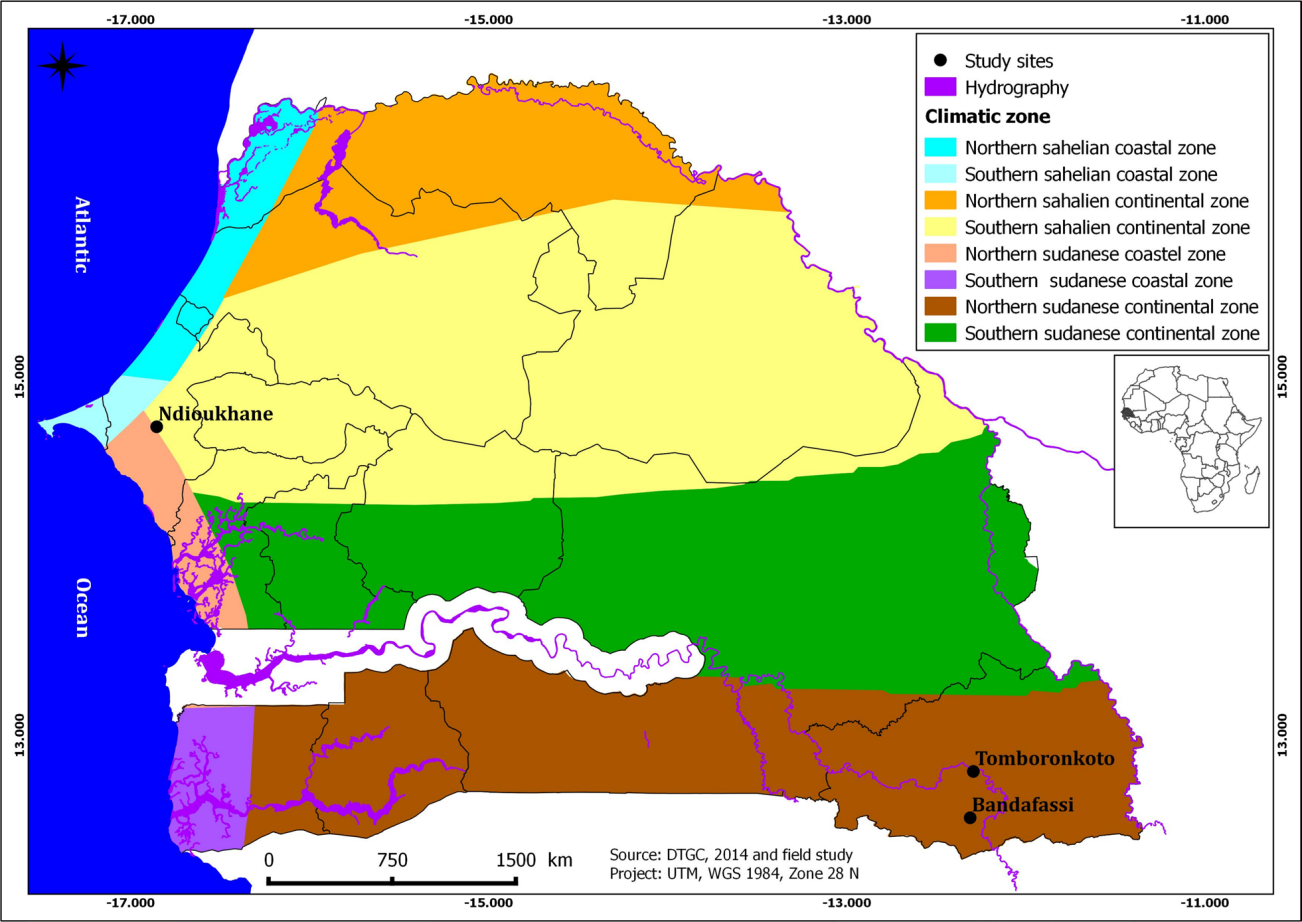
Geographical position of the experimental huts station (Ndioukhane) and the IRS sites (Tomboronkoto and Bandafassi) in Kedougou health district

The study was carried out at two main sites with different climatic environments. The Ndioukhane experimental huts field station located in the Sudano-Sahelian zone of Senegal served for SumiShield 50WG semi field trials between August 2017 and July 2018 (Fig. 1). Ndioukhane village belongs to the Thiès health district, an area of 6670 km^2^ and an estimated population of 2,221,097 [10]. The average temperature in this region is 32 °C, with the lowest values recorded between January and February and the highest between March and October (35 °C). The average annual rainfall ranges between 400 and 600 mm per year. The region’s proximity to the Atlantic Ocean coast allows year-round humid weather with an average relative humidity of 62% varying between a maxima of 87% and a minima of 37%.

The second study site is the health district of Kedougou, where IRS using SumiShield 50WG was carried out and monitored between June 2020 and February 2021, precisely in the sentinel villages of Tomboronkoto and Bandafassi. The region of Kedougou is bordered by the Republic of Mali in the East, the Republic of Guinea in the South and toward the North and West by the Tambacounda region. Kedougou is located in a Sudano-Guinean area, covering a surface of 16,800 km^2^ with an estimated population of 196,991 [10]. Temperatures are generally high, with maxima varying between 34 °C and 42 °C (from March to June) and minima between 21 °C and 25 °C (from July to February). Relative humidity is high during the rainy season, where it exceeds 97% between August and October. It decreases from January to March reaching a minimum value of 10% [10]. Kedougou region is one of the rainiest in the country, with at least 1300 mm/year. The rainy season lasts about 6 months, from May to October, the months of August and September being the rainiest ones.

### Experimental hut trial

SumiShield 50WG is an IRS product formulation with clothianidin as the active ingredient. The formulation was tested at an application rate of (300 mg a.i./m^2^) on the cement vs the mud walls of the experimental huts. Hut treatments were carried out using the WHO-recommended compression sprayer (10 litters Hudson Xpert 67422 AD pumps (HUDSON Manufacturing Company, USA), fitted with a calibrated nozzle n°8002E. All sprayers were calibrated with water prior to treatment of huts. Before spraying, the operator carried out several tests on blank walls with a tank full of water to ensure that a uniform flow rate was established before started spraying.

The product was applied to a total of 6 huts, 3 of which were made of mud and 3 of cement. Two negative controls, sprayed with distilled water only, were also included.

The residual efficacy of the treatment was assessed one-week post-treatment, then every one month for up to 12 months. The susceptible strains of *Anopheles coluzzii* (a susceptible laboratory-reared *An*. *coluzzii* colony originating from Cameroon was maintained for several years, and its susceptibility was checked before each study) used to assess residual efficacy were raised at the insectarium in Dakar (UCAD) and Thies (SLAP), the transported to the field in adult emergence cages. Bioassays were carried out according to WHO standards tests procedures on the treated wall surfaces [11]. In each of the treated and untreated huts, four cones were taped to each of the four inner walls of the huts at varying heights from wall to wall. Ten (10) not blood-fed mosquitoes, aged 3–5 days, were introduced into each cone and exposed to treated walls for a 30 min; then transferred into cups labelled with the corresponding information of each cone at the end of the exposure. The observation cups were maintained at a temperature of 27 ± 2 °C, a relative humidity of 80 ± 10% and mosquitoes fed with a 10% sugar solution. Mosquito mortalities were recorded at 24 h, 48 h, 72 h and 96 h. The ambient temperatures and humidities were monitored throughout the whole period of the test.

### Wall cone bioassays in the treated district of Kedougou

The cone bioassays were carried out in 5 randomly selected treated rooms and one untreated control room in the two villages (Tomboronkoto and Bandafassi) following the standards WHO procedure [11]. An insectary-reared *An. coluzzii* strain maintained in the LEVP laboratory was used for the residual efficacy bioassays. At each time point, three cones were attached to the walls and ceiling of the IRS-treated surfaces at different wall heights (0.5 m; 1 m and 1.5 m). Three to five days old mosquitoes were transferred into cones in 3 batches of 10 and exposed to the treated surfaces for 30 min. As a control, mosquitoes were exposed into four (4) cones attached at different heights of untreated walls and ceiling of an unsprayed room. At the end of exposure, mosquitoes were transferred to netted plastic cups. Mosquitoes were provided with 10% glucose solution and kept at a temperature of 27 ± 2 °C and at a relative humidity of 80 ± 10. Twenty-four hours post-exposure observations were performed to record and count dead mosquitoes to estimate the delayed mortality, 48 h, 72 h and 96 h post-exposure. The same selected rooms and position where cones were taped on the wall chosen were kept throughout the surveys. Before data interpretation, the test was validated and corrected with the Abott’s formula when the mortality observed in the controls was between 5 and 20%. When the mortality rate in the controls exceeds 20%, the test was discarded then repeated [11].

### Data analysis

Data were entered into a Microsoft Excel spread sheet and analyses were performed with the R 3.6.1 software version. Treatment residual efficacy was assessed according to WHO criteria by comparing the mortality rates observed at the 80% threshold. The X-squared test or the exact Fisher test were used when appropriate to compare Knock down Time and mortality rate. All tests were performed at the 5% significance level.

## Results

### Residual efficacy of SumiShield 50WG under experimental huts semi-field conditions

**Fig. 2:**
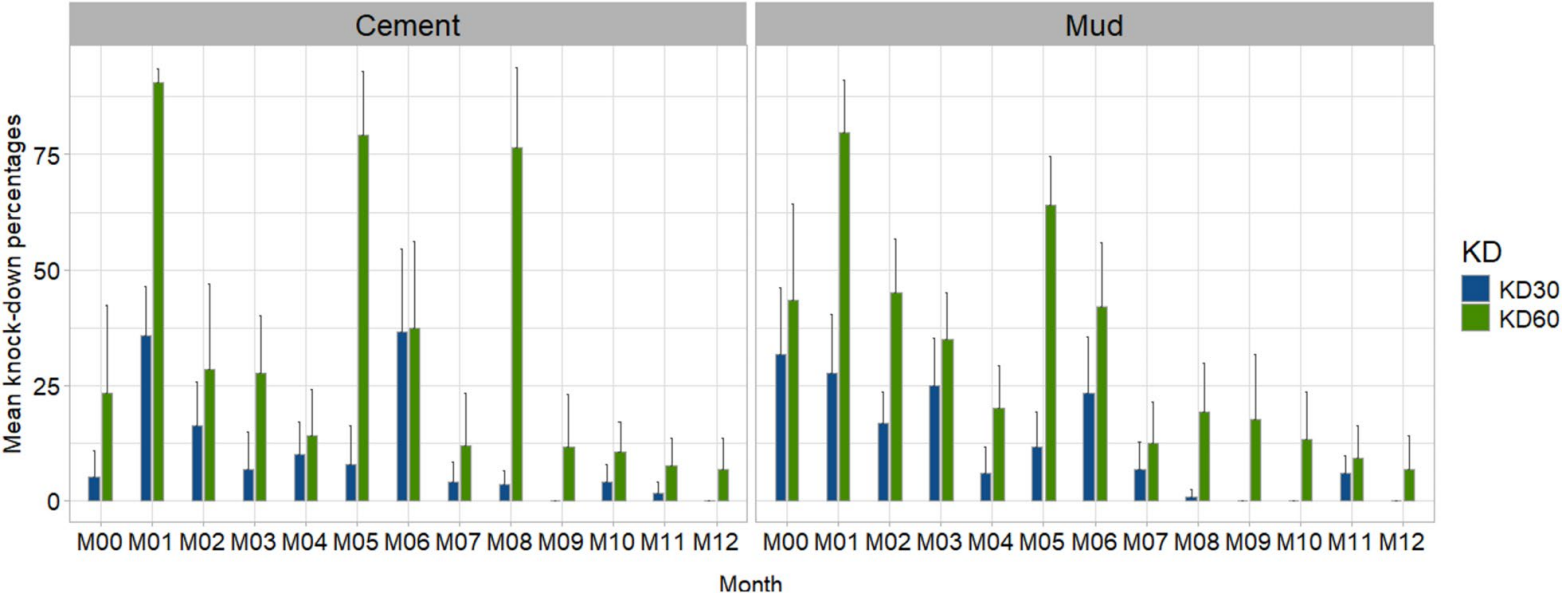
Knock down 30- and 60-min post-exposure of the SumiShield 50WG formulation on the cement and mud walls of the experimental huts

The overall results of the experimental huts trial, 96 h post-exposure, showed that the SumiShield 50WG formulation has a residual efficacy up to the 12 months of the survey with mortality rates exceeding the standard WHO threshold of 80% both on mud and cement walls. On the other hand, the knock down rates 60 min post-exposure were below 50% whatever the wall types and whatever the survey month, except for months 1 and 5 for mud and cement walls, and month 8 for cement walls (Fig. 2).

**Fig. 3:**
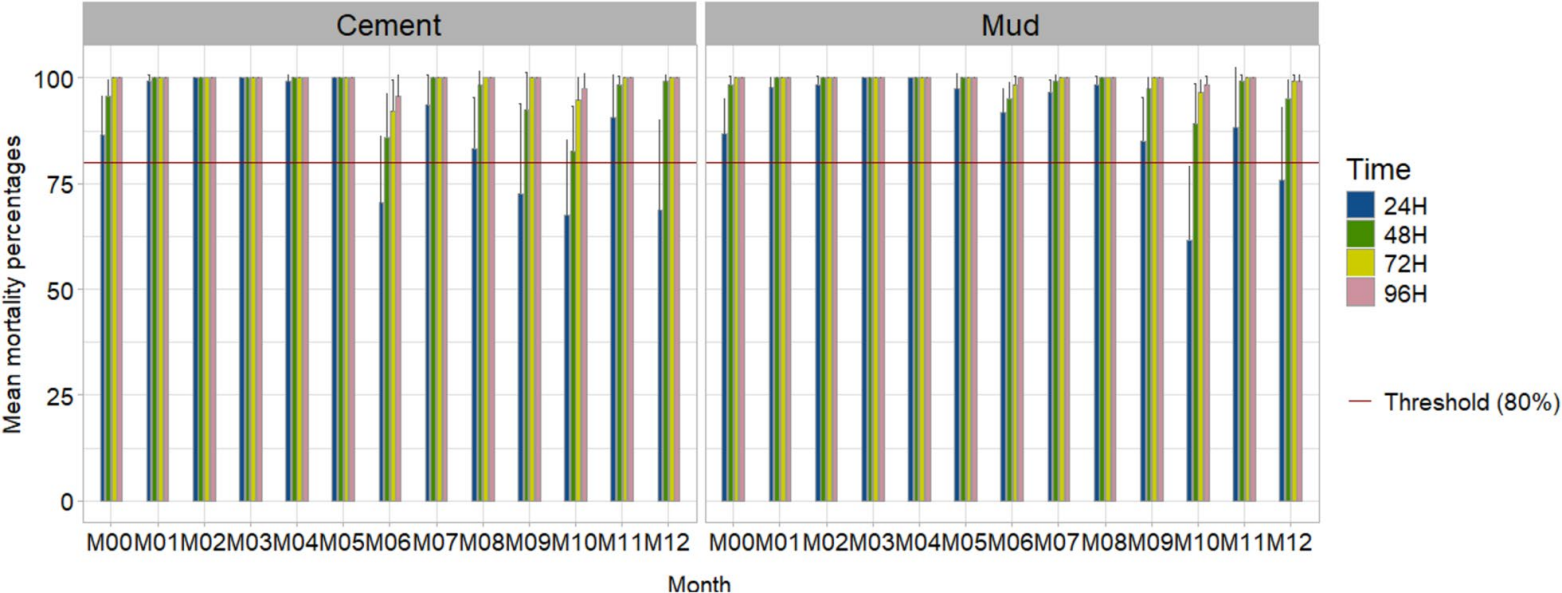
Mortality rates 24, 48, 72 and 96 h post-exposure of the SumiShield 50WG formulation on the cement and mud walls of the experimental huts

Over the study period, the SumiShield 50WG formulation induced high mortality rates, above 80% for all wall types. More specifically a mortality rate of 100% was recorded 48 h and 72 h post-exposure, excepted the month 6 for cement and mud with a mortality rate of with 92.2% and 98%, respectively (Fisher’s exact test: OR 5.05; 95% CI 1.01–49.12, *P* = 0.030); and the month 10 with respective rates of 95%, 97% (Fisher’s exact test: OR 1.05; 95% CI 0.34–49.12, *P* = 7.47) (Fig. 3).

### Residual efficacy of SumiShield 50WG under operational field conditions in Kedougou

**Fig. 4:**
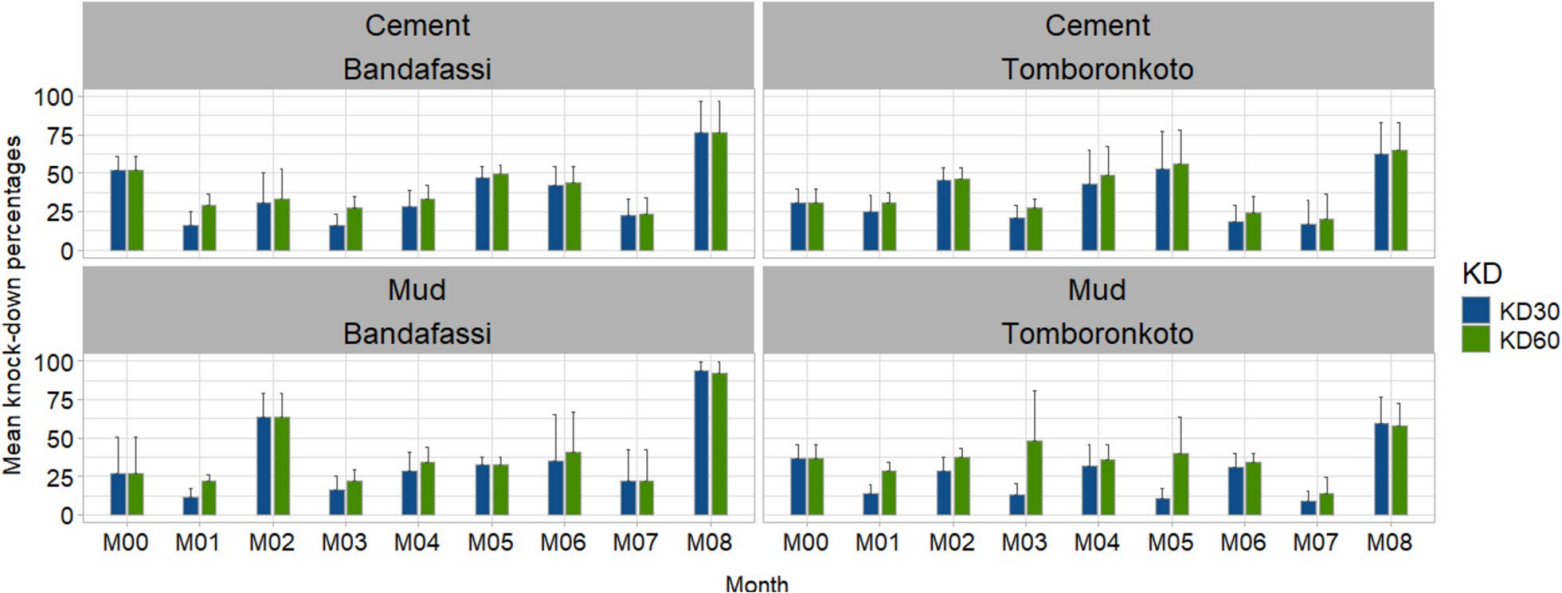
Knock down 30- and 60-min post-exposure of the SumiShield 50WG formulation on the cement and mud walls of the treated habitations in Tomboronkoto and Bandafassi

**Fig. 5:**
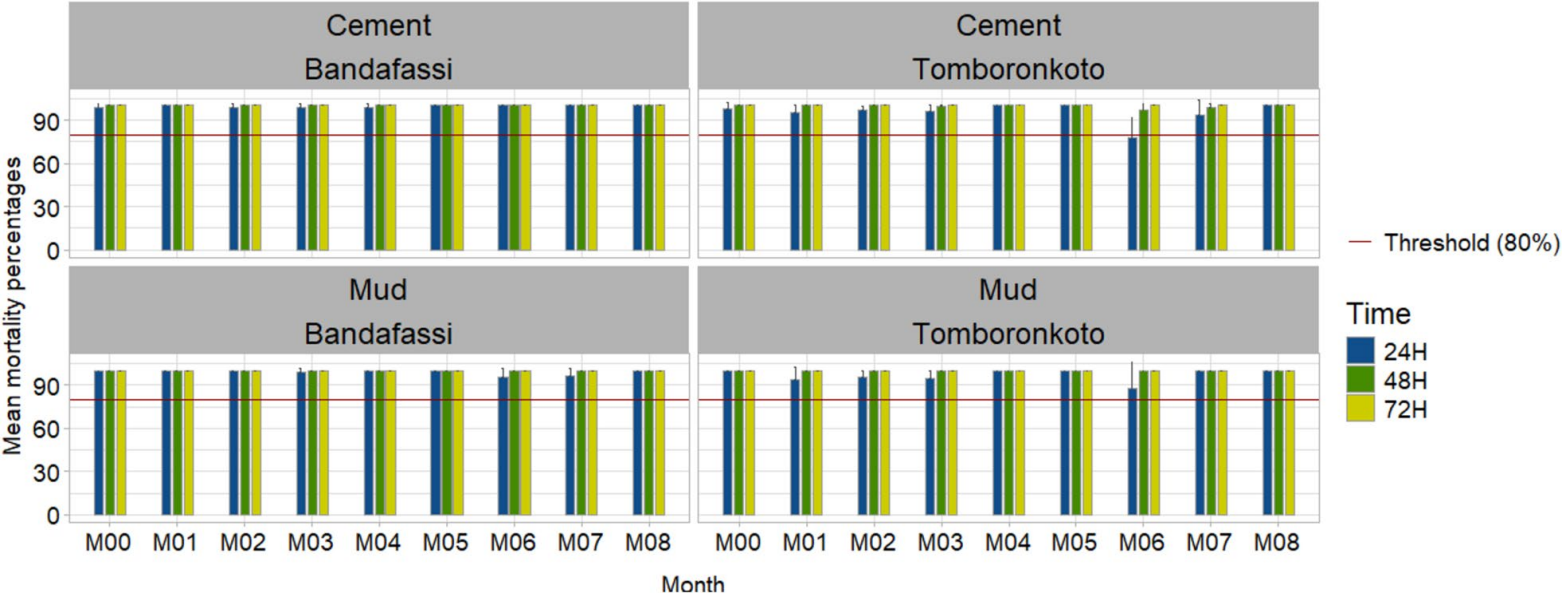
Mortality rate after 24 h, 48 h, 72 h post-exposure of the SumiShield 50WG formulation on the cement and mud walls of the treated habitations in Tomboronkoto and Bandafassi

The residual efficacy of the SumiShield 50WG formulation was monitored in the villages of Tomboronkoto and Bandafassi in the Kedougou health district. The 60 min-knock down rates in Bandafassi for all months up to the month 8 both for the cement walls (X-squared = 75.89, df = 1, *P* < 0.0001) and the mud walls (Fisher’s exact test: OR 0.012; 95% CI 0.001–0.054, *P* < 0.0001) (Fig. 4). The 72 h post-exposure mortality rate remained above 80% up to the month 8 both on the cement and mud walls. While the 60-KD time and the 72 h post-exposure mortality rates showed full effectiveness up to 8 months post-treatment on *An. coluzzii* susceptible strain on the two walls type both in Tomboronkoto and Bandafassi. However, the 24 h post-exposure of the SumiShield 50WG formulation was above 80% over the 8 months post-treatment in Bandafassi and Tomboronkoto, then reached 100% 72 h post-exposure during the whole survey period (Fig. 5).

## Discussion

Climate change significantly influences the geographic distribution and epidemiology of malaria [12, 13], which transmission is closely influenced by the rain regimen as well as the rainy and the dry seasons. This is specifically true in the Sahelian countries such as Senegal, where the rainy season lasts up to 5 months between June/July and October [14]. Under these conditions, to effectively protect population and cover the transmission period, the IRS insecticide formulations must have a residual efficacy beyond the transmission season or require two treatment cycles, which presents considerable economic and material limitations [15].

The widespread of resistance to organochlorine, pyrethroids, organophosphates and carbamates families of insecticides threatens the success gained so far, jeopardizing the elimination goal [16]. To overcome the above-mentioned challenges third generation insecticide for IRS (3GIRS) such as the neonicotinoids was proposed as alternatives to control pyrethroid-resistant mosquitoes [17].

Insecticide resistance cannot be managed by replacing one insecticide with another of the same mechanism of action, but rather by considering key parameters of mosquitoes carrying resistance genes, avoid exposing mosquitoes to the same product for several years, and so on [18]. Other strategies, such as the rotation of insecticides with different modes of action for indoor residual spraying and impregnation of mosquito net have been recommended to improve malaria vector control. Amongst the new insecticides evaluated for IRS, the neonicotinoids, clothianidin, has been identified as a potential alternative for insecticide resistance management. Despite the gradual emergence of resistance to clothianidin in *Anopheles gambiae* populations in some agricultural areas, linked to the intensive use of agrochemicals belonging to the neonicotinoid class for crop protection [19, 20], given the low probably for cross-resistance with other IRS insecticidal classes currently in use, while generally exhibiting very low mammalian toxicity [21].

The results of our study showed good residual efficacy of SumiShield 50WG both on the mud and cement substrates, either in the experimental and operational conditions, with delayed mortality rates above the WHO threshold. The monitoring of the residual efficacy under experimental huts conditions showed 12 months post-treatment effectiveness as showed by the delayed mortality rates 24 h, 48 h and 72 h post-exposure. While the results from operational IRS in the study villages showed full efficacy 8 months post-treatment. The results corroborate several previous studies elsewhere in Africa [17, 21, 22].

The immediate 24 h post-exposure mortality under experimental huts, which mimics semi-natural, showed a full residual efficacy of the SumiShield 50WG formulation on cement substrates up to 8 months, with the exception the 6th month below the WHO threshold (80%). The same trend was observed in the village of Tomboronkoto on the cement walls. The unexpected decrease in the mortality rate of mosquitoes exposed to cement surfaces at the sixth month, both in experimental huts and in the field, could be explained by a possible relapse of the insecticide in the treated walls. This phenomenon could be linked to climatic or environmental factors or to the mechanisms of delayed release of the insecticide, several months after the initial application. On the other hand, the mud walls showed mortality rates above the WHO threshold up to 11 months post-treatment under experimental conditions; and up to 08 months under operational conditions in the IRS-villages monitored. These results corroborate with the studies by [21] and [17].

The study has certain limitations, particularly those related to the COVID-19 pandemic. Health restrictions, strict protocols, and public mistrust made it difficult to carry out certain tasks and to travel long distances sometimes several hundred kilometres. Additionally, the slow-acting effect of clothianidin on mosquito mortality (delayed mortality), combined with its lack of noticeable odour, led some residents to believe that the product was attracting mosquitoes and was responsible for an apparent increase in their presence. Furthermore, some households repainted their walls before the study ended, which may have affected the results.

## Conclusions

The study showed that the SumiShield 50WG formulation displays good residual efficacy up to 12 months under experimental conditions and up to 8 months in the natural field conditions on all treated and tested substrates. The use of the 3GIRS of the neonicotinoid family, namely clothianidin, to which no resistance has been reported so far from malaria vectors in Senegal, has been recognized as a good tool for insecticide resistance management through rotation strategy of insecticides with different modes of action between spray rounds.

## Data Availability

Data supporting the conclusions of this article are included within the article. The data used and analyzed during the current study are available from the corresponding author upon reasonable request.

## Abbreviations

WHO: World Health Organization
IRS: Indoor residual spraying
ANSD: Agence national de la statistique et de la démographie
LLINs: Long-lasting insecticide-treated nets
NMCP: National Malaria Control Programme
UCAD: Université Cheikh Anta Diop
SLAP: Service de Lutte Antipaludique
OR: Odd ratio
P: Probability
KD 60 min: Knock down 60 min
PQT-VC: Prequalification team-vector control products
ITNs: Insecticide-Treated Nets
3GIRS: Third-generation indoor residual spraying
